# Lithium phthalocyanine (γ-structure) as a molecular oxygen sensor†

**DOI:** 10.1039/d4ra08335k

**Published:** 2025-02-05

**Authors:** Elena Tomsik, Zulfiya Cernochova, Magdalena Scheibe, Krzysztof Tadyszak

**Affiliations:** a Institute of Macromolecular Chemistry, Czech Academy of Sciences Prague Czech Republic tadyszak@imc.cas.cz cernochova@imc.cas.cz; b J. Heyrovsky Institute of Physical Chemistry, Czech Academy of Sciences Prague Czech Republic

## Abstract

Synthesis and characterization of lithium phthalocyanine radicals were performed, which were followed by an investigation on its ability to detect oxygen levels in biologically relevant concentrations. EPR studies confirmed the presence of at least two phases, one sensitive to oxygenation (γ-phase) and the second one insensitive to oxygenation (α-phase). Contrary to the findings reported in the literature, it was observed that the γ**-**phase was not stable at and above 95 °C and slowly transformed into the α-phase crystallographic structure. Above 150 °C, only a broad signal of α-phase existed. Additional characterizations were performed using electrochemical impedance spectroscopy (EIS), cyclic voltammetry, dynamic light scattering (DLS), and Raman spectroscopy on pristine crystals Li_2_Pc and LiPc sensors.

## Abstract 2

Synthesis and characterization of lithium phthalocyanine radicals were performed, which were followed by an investigation on its ability to detect oxygen levels in biologically relevant concentrations.

## Graphical abstract

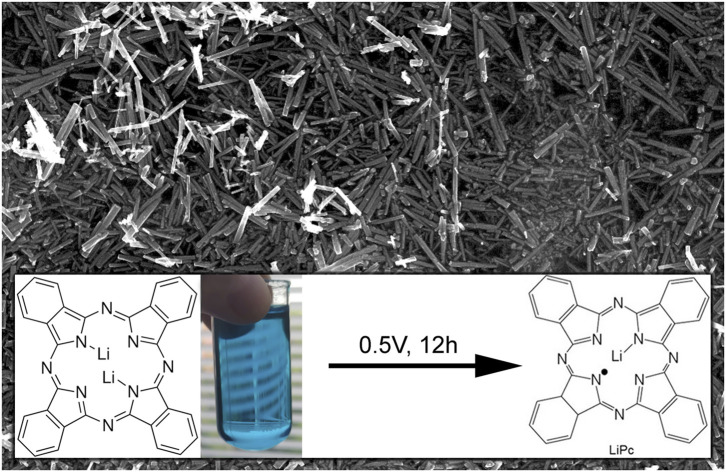

## Introduction

Changing oxygen levels in the body can be a marker of many diseases, *e.g.*, inflammatory processes and tumor formation.^1^ Applied treatments are also dependent on the oxygenation levels of the treated region. Hence, oxygen sensors are essential for biological studies in various tissues, tumors, 3D cell cultures, and single cells.^1,2^ Sensors applying electron paramagnetic resonance as a method of choice for radical detection^3,4^ and oximetry^5^ would be beneficial for bio/medical fields. Lithium phthalocyanine sensors are semiconducting microcrystalline sensors with the unique feature of a narrow peak-to-peak linewidth without oxygen that varies slightly as reported in the literature, *e.g.*, 10–70 mG.^2,6–10^ A very narrow signal in the EPR allowed the detection of a small quantity of material (spin concentration *N* = 9.2 × 10^19^ spins per g,^2^ 0.1–0.05 spins per molecule^10,11^). Hence, it is helpful as a sensor in EPR and NMR magnetometry.^12–14^ Magnetometers based on LiPc microcrystals are two orders more sensitive than Hall-probe magnetometers.^15^ These sensors can be a part of larger projects and microfluidic systems, *e.g.*, EPR-on-a-Chip^16^ and additional sensors for Oxy-chip.^17^ Even though LiPc can detect NO* radicals, it could not determine their *in vivo* levels (1–10 nM) in the presence of physiological concentrations of O_2_.^18^

The π-electron neutral radical of lithium phthalocyanine – LiPc (*S* = 1/2) derived from dilithium phthalocyanine (Li_2_Pc) is insoluble in water semiconductors in its crystalline phase.^19^ This compound can crystallize in various crystalline phases, α, β, and γ (also called x-phase), which show different properties (conductivity and magnetism, where α and β phases are ferromagnetic and antiferromagnetic, respectively). Their linewidths are in the ranges of Δ*B*_1/2α_ = 1−1.5*G*; Δ*B*_1/2β_ = 0.8−1*G*;^7^ Δ*B*_1/2γ_ = ∼0.01–0.6*G*,^18^ oxygen sensitive is only the γ-phase .^6,7,18^ LiPc is a chemically stable system in the temperature range of 0–200 °C;^7^ however, between the temperature range of 125–175 °C, both α and β phases coexist in various proportions.^7^ Literature shows that the γ-phase is stable up to 150 °C,^6,20^ and together with other parameters, it is a good sensor candidate for biology^21,22^ and medicine.^23–25^ It shows the following superior properties: (i) sensitivity to oxygen (5 G bar^−1^ (ref. 7)) owing to the presence of channels in which the dioxygen molecules can migrate; (ii) a substantial overlap between consecutive LiPc molecules in a stack and a very efficient spin diffusion revealed by a very narrow EPR line (Δ*B*_pp_ < 20 mG); (iii) O_2_ molecules interact only magnetically and not chemically with LiPc* and this process is reversible and shows linear concentration dependence in a broad range; (iv) biocompatible and stable in biological conditions for months, which makes them an ideal system for long-period oxygen level monitoring.^7,10,20,26^

In the most straightforward model, molecular oxygen has two unpaired spins (*S* = 1) in the ground state, creating a magnetic field around itself. Oxygen sensing is based on detecting magnetic field fluctuations caused by O_2_ molecules. These fluctuations cause a broadening of the EPR line, which can be quantified according to oxygen concentration. This dependence is linear (5 G bar^−1^ (ref. 7)) for biological oxygen concentrations, *e.g.*, Δ*B* = 14 mG for 0% O_2_, and goes up to 1 G for 160 mmHg (21.3 kPa)^2^ (varies between the crystals).

The current work was initiated with the following goals: (1) synthesis of lithium phthalocyanine radicals with controlled morphology; (2) it was proved that γ-phase is unstable in and above 95 °C and slowly transforms into α-phase; (3) extensive characterization of LiPc radicals by Raman spectroscopy, dynamic light scattering and zeta potential measurement, cyclic voltammetry and electrochemical impedance spectroscopy, and EPR line-shape analysis; (4) evaluation of LiPc crystals ability to detect oxygen level at biological relevant concentrations.

## Experimental

### Synthesis

**Fig. 1:**
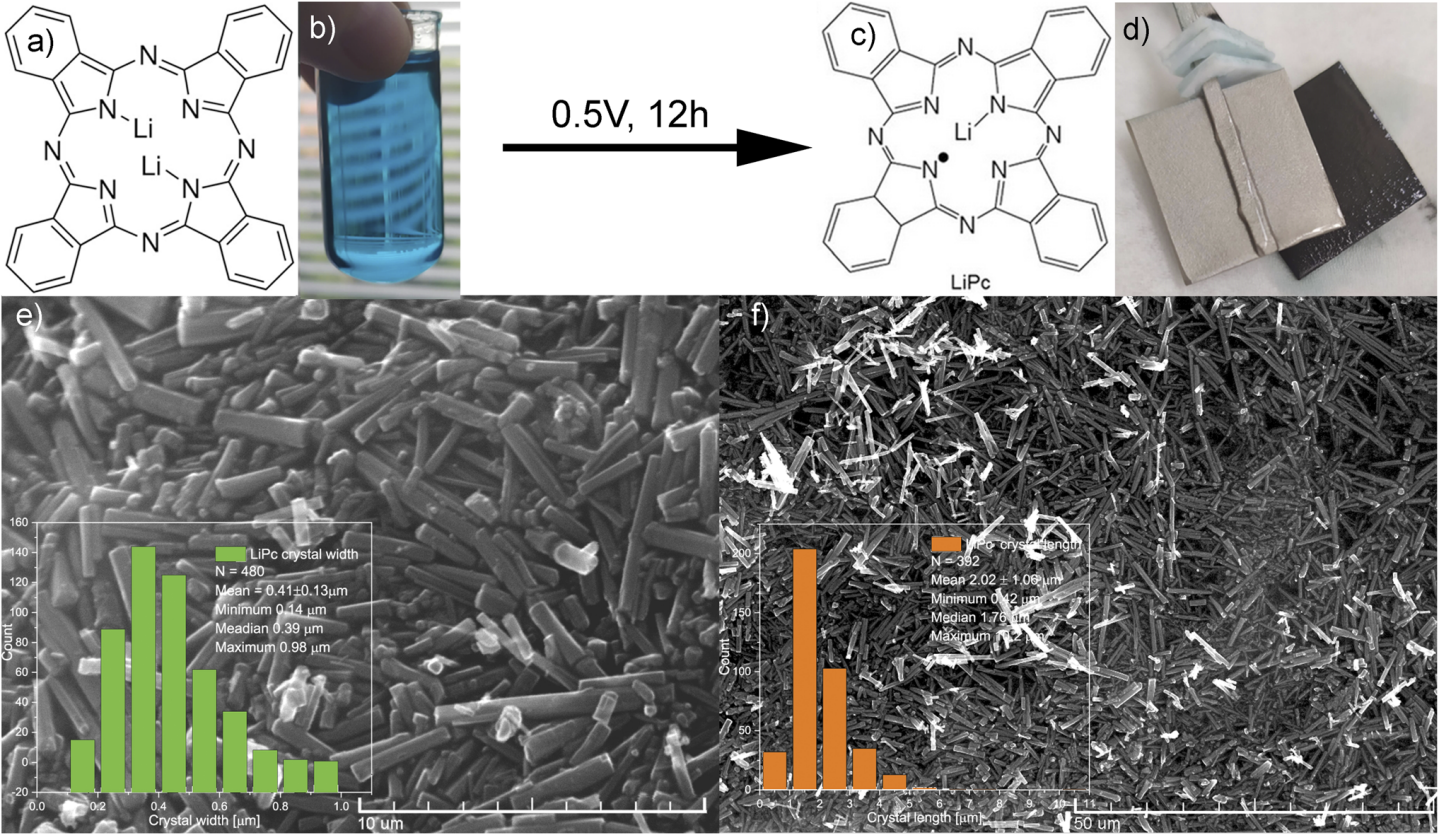
(a) Chemical structure of dilithium phthalocyanine–Li_2_Pc; (b) diluted Li_2_Pc in acetonitrile; (c) chemical structure of lithium phthalocyanine – LiPc; (d) platinum electrodes with LiPc (black layer) on one of them; (e) SEM image of γ-LiPc – scale 10 μm, inset – crystal width statistic; and (f) SEM image of LiPc – scale 50 μm, inset – crystal length statistics.

Lithium phthalocyanine γ crystallographic structure (γ-LiPc, Fig. 1c and d) was obtained by electrochemical synthesis from dilithium phthalocyanine. Li_2_Pc (50 mg, Fig. 1a) was dissolved in 30 ml DMSO using ultrasonication (15 min). Then, the solution was dried and once again dissolved in 30 ml of dried acetonitrile (Fig. 1b) without the addition of tetrabutylammonium perchlorate (TBAP),^19,27^ or tetraethylammonium perchlorate (TEAP)^28^ as mentioned in the literature previously. The setup consisted of two platinum electrodes, 4 cm^2^ each, separated by a Teflon layer. Before synthesis, platinum electrodes and a Teflon separator were immersed in a 65% nitric acid solution for 30 minutes, after which they were rinsed with water and acetone, dried, and used for synthesis. Electrosynthesis was performed overnight without stirring at room temperature (12 h) at 0.5 V (OWON ODP3031 Power Supply). After synthesis, a thin layer of crystals appeared on the positively polarized electrode. The electrode was washed with water, and crystals were collected with cotton and placed in a quartz X-band EPR tube. After multiple syntheses, the minimum linewidth peak-to-peak for 0% O_2_ was in the 35–65 mG range for various samples. Samples were continuously measured at a standard pressure of 101 325 Pa in various gas mixtures, air, and O_2_/N_2_ mixtures. Conversion from oxygen partial pressure in mmHg to % is as follows: 21% O_2_ is 21 278.25 Pa (159.6 mmHg).

### Electron paramagnetic resonance (EPR)

The EPR measurements were conducted with a Bruker ELEXSYS 540 X-band spectrometer with a 049× microwave bridge (Super X) and a Bruker ER4108 TMHS slim design resonator. The following EPR spectrometer settings were applied for the experiments: case 0% O_2_-narrowest EPR line-microwave power: 20.17 μW (40 dB), modulation amplitude 0.01 G; modulation frequency: 10 kHz; the time constant: 5.12 ms; conversion time: 20.48 ms; gain: 55 dB; the number of points: 2048, temperature 294 K, 1 accumulation; case ≥21% O_2_-parameters were as follows: microwave power: 2.012 mW (20 dB), modulation amplitude 0.1 G, gain: 60 dB (other parameters identical). The *g*-factor of the internal standard Al_2_O_3_:Cr^3+^ placed at the second resonator's entry was calibrated by comparison with DPPH radical (*g* = 2.0036). The internal standard remained in the resonator during all the measurements.

### Electrochemical deposition of LiPc film

The LiPc film was electrochemically deposited using Metrohm® AUTOLAB Potentiostat/Galvanostat PGSTAT302N with a frequency analyzer FRA32M module controlled by the software NOVA 2.1.4., at the Pt electrode by a cyclic voltammetry method in a three-electrode cell configuration. The Li_2_Pc was dissolved in dried acetonitrile. Two potential windows were applied: (a) from −0.3 to 1.2 V *vs.* Ag/AgCl wire and (b) from −0.2 to 1 V *vs.* Ag/AgCl wire. The nitrogen gas was purged above the solution to protect it from oxygen dissolution. 500 cycles were conducted with a scan rate of 50 mV s^−1^.

### Cyclic voltammetry measurement of LiPc film

The electrodes were characterized in three-electrode cell configurations. All measurements were done at room temperature. A Pt sheet was employed for the counter electrode, and Ag/AgCl (3 M KCl) was used for the reference electrode. Cyclic voltammetry was conducted in acetonitrile with tetrabutylammonium perchlorate solution between −0.2 and 0.9 V *versus* an Ag/AgCl reference electrode with scan rates from 10 and/or 150 mV s^−1^.

### Electrochemical impedance spectroscopy (EIS)

A Metrohm® AUTOLAB Potentiostat/Galvanostat PGSTAT302N was used to perform EIS with a frequency analyzer FRA32M module controlled by the software NOVA 2.1.4. In acetonitrile with tetrabutylammonium perchlorate solution at the open circuit potential (OCP) over a 100 kHz to 0.01 Hz frequency range with 5 mV amplitude. The equivalent circuit analysis was completed, and the chi-square (*χ*^2^) value points out the divergent regions between the fitted and measured data.

### Raman spectroscopy

Ambient Raman spectra were recorded using a WITec Alpha300R spectrometer of 600 lines per mm grating equipped with a piezo stage (200 × 200 × 20 μm) and a RayShield Coupler with laser power of 300 μW (532 nm and 633 nm excitation wavelengths) for all measurements. Raman measurements were conducted at room temperature (298 K) under ambient conditions. To prevent photodegradation, the samples were scanned with minimal laser exposure time and low power settings. Lithium phthalocyanine crystals were transferred and measured by exfoliation onto Si/SiO_2_ wafers. Before using the Si/SiO_2_ wafers, they were cleaned in the ultrasonic bath for 5 min in EtOH, 5 min in IPA, and then in oxygen plasma for 1 min. The Raman bands were assigned based on previous literature and confirmed by comparison with standard spectra of phthalocyanines.

### Dynamic light scattering (DLS) and zeta potential measurements (*ζ*)

We measured the intensity-weighted hydrodynamic radius (*R*_H_) and scattering intensity and *z* of the lithium phthalocyanine particles in the water solution on a Zetasizer NanoZS instrument, model ZEN3600 (Malvern Instruments, Malvern, UK) at the temperatures of 25 °C and 37 °C.^29^*R*_H_ was measured at a scattering angle of *θ* = 173°, and the data were processed with the Malvern software.^30^ The surface zeta potential of silica was measured using a Malvern ZEN1020 surface zeta potential cell. The cell consists of a sample barrel with adjustable height, where the sample is placed on a holder and held between two electrodes made of palladium. A series of measurements of the zeta potential was then performed in a standard cuvette, and the position of the measurement was controlled by adjusting the height of the sample holder. Lithium phthalocyanine particles of a particular type were used as tracer particles for appropriate lithium phthalocyanine crystals layer glued into the copper surface (thickness 0.3 mm). The zeta potential data were evaluated using the Smoluchowski model.^31,32^

## Results and discussion

According to the literature data, a multi-phase composition of the LiPc crystals is usually obtained in one batch. However, one of the goals of the current work is to synthesize only the γ-phase crystallographic structure due to its oxygen sensitivity, in contrast to the non-sensitive α-phase. The β-phase appears after heating the α-phase crystals above 400 °C,^7^ a conditions which is not followed in this work. Reducing potentials are usually in the range of 0.05–0.75 V.^28^ Furthermore, it was suggested that the oxygen must be removed from the solution before synthesis. However, we found no benefits of this additional step. This work utilizes EPR spectroscopy as the primary method of evaluating synthesis outcomes and sensor quantification. Due to its sensitivity and resolution, it is possible to study small powder quantities obtained after synthesis, which is usually insufficient for powder XRD. Due to the high resolution of EPR spectroscopy, small phase changes can be observed. The minimal EPR linewidth obtained for 0% and 21% O_2_ are generally good indicators of system purity, and unfortunately, both can enormously vary from synthesis to synthesis. We performed electrochemical synthesis using two platinum electrodes of *ca.* 2 cm^2^ each at 0.5 V overnight-12 h in ambient conditions (Fig. 1a–d). The dilithium phthalocyanine dissolved in acetonitrile is of blue color (Fig. 1b), which dilutes/fades during the crystal growth on the positively polarized electrode (Fig. 1d, black crystals). The SEM micrographs presented in Fig. 1e and f show needle-type crystals, as mentioned in the literature.^12,19,20,33^ Statistical analysis performed over multiple SEM images shows an average crystal length of 2.02 ± 1.06 μm (number of measurements 392, min. 0.42, median 1.76, max. 10.24 μm) while the width on average is 0.41 ± 0.13 μm (number of measurements 480, min. 0.14, median 0.39, max. 0.98 μm). Histograms of this study are shown as insets in the exact figure.

### Dynamic light scattering

**Fig. 2:**
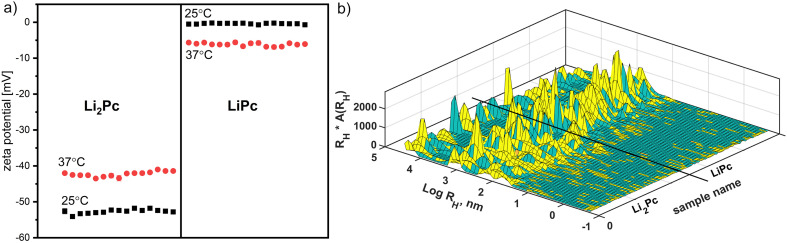
(a) Multiple zeta potential measurements for Li_2_Pc and LiPc water solutions at 25 °C and 37 °C; (b) repeated DLS measurements for Li_2_Pc and LiPc samples at 25 °C.

Lithium phthalocyanine microcrystals are intended for biological systems. It is essential to know their electrokinetic parameters. In this study, Li_2_Pc—original and after-synthesis LiPc crystals were studied. As a tracer particle, the upper part of the water solution was used to add the crystals of Li_2_PC and LiPc. These quasi-solutions were then centrifuged, and the upper part was collected. Dynamic light scattering (DLS) and zeta potential (*ζ*) measurements revealed the random Brownian motion in these solutions. The results are shown in Fig. 2. The measurements were performed at room and physiological temperatures (37 °C), as indicated by black squares and red circles, respectively. The morphology of the crystals changes due to the synthetic conditions used for preparation, and one lithium-ion dissolves from the structure. This causes the zeta potential to increase to zero values. Moreover, this trend remains with temperature changes.

As shown above, the sample with one lithium-ion is more suitable for biosensor applications than Li_2_Pc, as it has a lower electronic capacity and a lower charge in zeta potential measurements. This suggests that a lower-charged surface would be less irritating to the immune system.

The tracer particle sizes, expressed in Table 1, were measured simultaneously with zeta potential. With increasing temperature, the values of the Li_2_Pc sample may change slightly due to the self-assembly but remain comparable. The size of the LiPc sample in the tracer solution remains constant. The measurements yielded reproducible and stable results over time, so they were selected for further analysis. Additionally, it can be seen from the table that the conductivity of the water solution was significantly higher in the Li_2_Pc sample, which may be due to the nature of the dissolved particles.

**Table 1:** Results of DLS and electrophoretic measurements of tracer solution and layers of Li_2_Pc and LiPc samples

Sample	Number mean size *R*_H_ [nm]	Zeta potential [mV]	Mobility [μm cm V^−1^ s^−1^]	Conductivity [mS cm^−1^]	Surface zeta potential [mV]
**25 °C**					
Li_2_Pc	115.96 ± 16.08	−52.68 ± 0.58	−4.02 ± 0.14	1.36 ± 0.041	−53.08 ± 2.37
LiPc	282.25 ± 80.56	−0.4 ± 0.16	−0.026 ± 0.012	0.041 ± 0.005	−2.4 ± 0.61

**37 °C**					
Li_2_Pc	310.77 ± 66.61	−42.27 ± 0.73	−4.08 ± 0.05	1.37 ± 0.03	−43.59 ± 1.81
LiPc	266.48 ± 41.59	−6.15 ± 0.44	−0.51 ± 0.13	0.0755 ± 0.0015	−7.82 ± 1.52

The thickness of the double electrical layer (*κ*^−1^) depends on the concentration of the ions in the solution and can be calculated based on the ionic strength of the medium. The greater the ionic strength, the more compact the double layer becomes. The valence of the ions also affects the thickness of the double layer. A divalent ion, such as Li^2+^, will compress the double layer more than a monovalent ion, such as Li^+^. As all measured solutions were prepared using deionized water, we would not expect such a high conductivity parameter (Table 1). We also selected smaller particles because the samples were centrifuged, and the upper part was chosen for measurements. Consequently, the samples demonstrated more populations (Fig. 2b). The MATLAB scheme in Fig. 2b presents dynamic light scattering measurements of both samples at room temperature. Sample LiPc showed lower polydispersity than Li_2_Pc. The latter sample has smaller particles, around 10 nanometers and less. They exhibit faster kinetics and are probably responsible for the higher conductivity of the Li_2_Pc solution.

The sensitive surface of the biosensor prototype was created by gluing large crystals of a particular type onto a copper plate approximately 0.3 millimeters thick. Since the synthesis of LiPc and Li_2_Pc crystals was performed on a platinum plate and held well on this surface, we selected the thin metallic plate made from copper for surface zeta potential experiments. The glued layer of lithium phthalocyanine onto the copper surface was black, and the thickness of the plate was slightly changed.

As expected, the surface zeta potential measurement results in Table 1 demonstrate the same trend as the tracer particles. To ensure the correct repulsion between the wall surface and the tracer particles, tiny crystals were used as a tracer solution, and large crystals were held onto the copper surface.

As the tracer solution of Li_2_Pc showed a high negative zeta potential, the experiment demonstrates that the wall is composed of the sa me type of larger crystals, which are equally charged in the vicinity with experimental accuracy. The lower-charged LiPc particles showed a movement closer to the wall covered with larger LiPc particles, expressed by the appropriate repulsion. With increasing temperature, the surface *ζ* potential of the LiPc layer decreased to negative values, but the tendency between the two samples remained at higher temperatures.

### Raman scattering

**Fig. 3:**
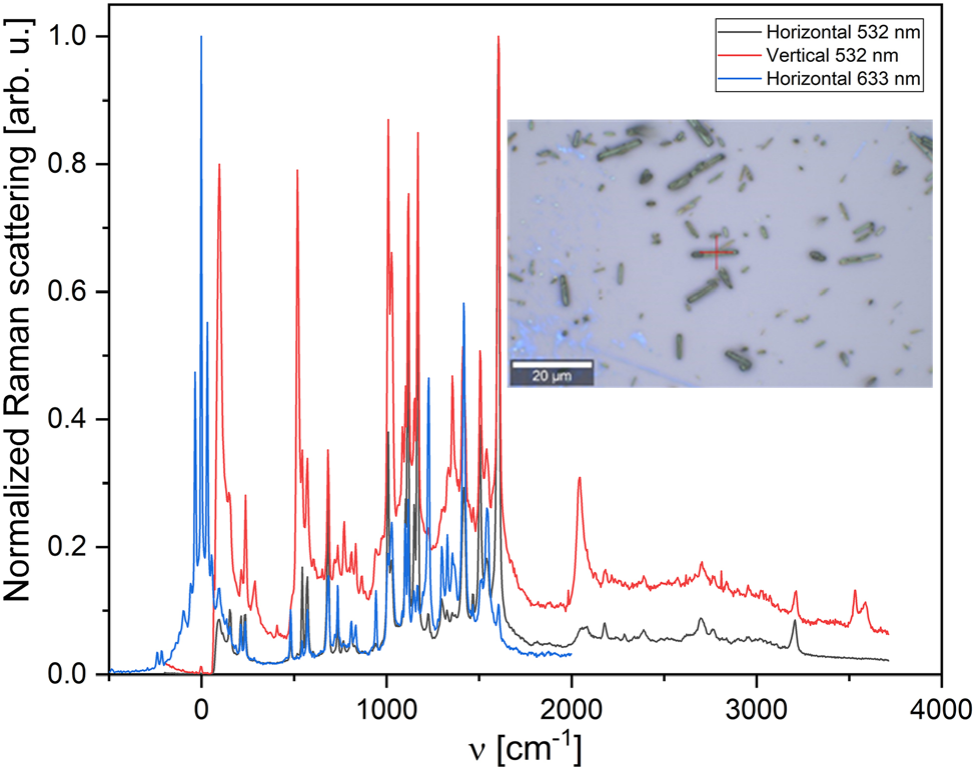
Horizontal and vertical Raman spectra of γ-LiPc crystals; inset shows an optical microscope image of the crystal.

LiPc is known for its distinct Raman peaks, which can be used to identify and analyze. The Raman spectrum of LiPc includes several characteristic peaks, particularly in the regions influenced by the vibrations of the phthalocyanine ring and the interactions with the lithium-ion. Some significant Raman peaks for LiPc typically occur in the following areas: in the range of 680–690 cm^−1^ (C–N–C ring stretching), in the range of 1120–1140 cm^−1^ (in-plane deformation of the macrocycle), in the range of 1300–1350 cm^−1^ (C–H bending), in the range of 1500–1600 cm^−1^ (ring breathing modes). These peaks indicate the structural and vibrational properties of lithium phthalocyanine. They can vary slightly depending on the experimental conditions and the form (thin film or bulk material) in which the LiPc is studied (Fig. 3).^34^

The characteristic Raman peaks for dihydrogen phthalocyanine (H_2_Pc) are observed at 1540 cm^−1^ (CNC group stretch, macrocycle alteration), 1449 cm^−1^, and 1425 cm^−1^ (isoindole and pyrrole stretches), 1339 cm^−1^ (macrocyclic breathing mode), 1141 cm^−1^ (isoindole breathing mode), 1113 cm^−1^ (pyrrole breathing mode), 794 cm^−1^ and 722 cm^−1^ (macrocycle ring deformation), 681 cm^−1^ (coordinated nitrogen atoms macro breathing vibration), 231 cm^−1^ and 132 cm^−1^ (macrocycle ring deformations).^35^ These differences reflect the structural and electronic effects of the substituent groups on the phthalocyanine ring (Fig. 3).^36^

### Electron paramagnetic resonance spectroscopy

The Smoluchowski equation describes the collision rate (frequency of collisions) between molecular oxygen and LiPc spins, which depends on the diffusion coefficients of both entities, oxygen concentration, and the distance between them.^13^ Due to this dependence, the linewidth is expected to be directly proportional to the oxygen concentration, which is valid for low oxygen concentrations. The basis of the line narrowing is the Heisenberg spin–spin exchange between molecular oxygen and LiPc spins. Line narrowing is explained in a dual-spin model,^13^ which describes two types of spins: A (mobile/diffusive spins) and B (fixed/trapped spins by oxygen molecules). Without oxygen molecules, spins A interact with themselves with high frequency (lifetime *τ*_A_ ∼ 0). This leads to extreme exchange narrowing^37,38^ of the line. In the presence of oxygen molecules, A-type spins are trapped and converted to B-type. Trapped spins cannot effectively exchange their spins with other A spins, lowering the exchange frequency and extending their lifetime (*τ*_A,B_ > 0), which is related to line broadening.^38^ The Heisenberg exchange interaction is described by spin–spin exchange integral (*J*), which is strongly dependent on the dimensionality (*n*), Δ*B* ∼ *J*^−*n*^ (one-dimensional *n* = 0.5 (ref. 6) when *J* = 0, Δ*B* = max.).^10,18^ In larger crystals, the exchange integral should be more significant and signal narrowing more efficient^10,18^ according to the relation, Δ*B*_pp_ = *K*〈l〉^*α*^, *α* = −0.36, where 〈l〉 is the average size of crystallite, and *K* is a proportionality constant.^10^ Additionally, it was shown that during EPR saturation experiments, three lines were detected, of which only one was sensitive to oxygenation, to which the theory mentioned above applies. The origin of the additional lines can be related to crystalline defects.^6^

**Fig. 4:**
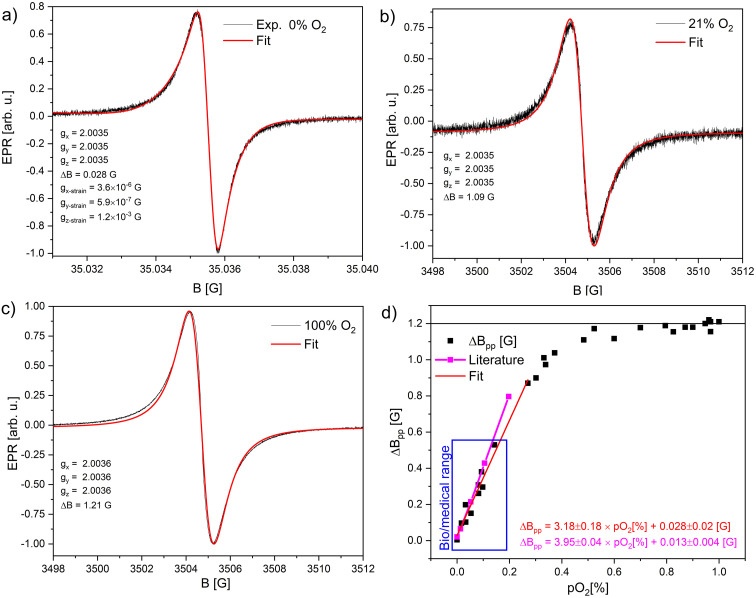
EPR spectra of LiPc: (a) saturated with N_2_; (b) in air; (c) saturated with O_2_; (d) peak-to-peak linewidth dependence *vs.* oxygen concentration of O_2_/N_2_ mixture. The dependence is almost linear and can be described as follows Δ*B*_pp_ [mG] = 3.18 × *p*O_2_ [%] + 0.028 in the range 0–21% of O_2_; this corresponds to the partial oxygen pressure range 0–159.6 mmHg or 0–21.3 kPa. The magenta line is the data from ref. 24 and 25.

Results presented here show a single narrow line (*ca.* 1 G at 21% O_2_-air), which is sensitive towards O_2_ molecules at atmospheric pressure (Fig. 4). After exchanging the air in the EPR tube with nitrogen, the narrowest EPR line obtained by fitting with Lorentzian function was 28 mG (Fig. 4a, Δ*B*_pp_ = 44 mG). Other samples showed similar peak-to-peak linewidth dispersion in the 44–65 mG range. The narrowest line saturates very quickly, for which measurements had to be performed at 40 dB attenuation, corresponding to 20.17 μW at room temperature. This is the lowest microwave power limit of Bruker spectrometers. Other technical parameters such as modulation amplitude and modulation frequency, which could generally broaden the linewidth, were also set to the lowest settings, correspondingly 10 mG and 10 kHz. Due to the very narrow line, the spectra accumulation artificially broadens the line due to the instabilities of the spectrometer. Fortunately, the signal was strong, and accumulations were not necessary. Fig. 4 shows EPR spectra and fits of one of the obtained samples for three cases of O_2_ saturation, 0%, 21%, and 100%. The sample was at atmospheric pressure for 0% and 100% in a constant 1 bar gas, nitrogen, or oxygen flow. Open-air conditions covered the 21% of oxygen saturation. Fig. 4d shows how the peak-to-peak linewidth depends on increasing oxygen content in the N_2_/O_2_ mix flow, which can fit linear equation Δ*B*_pp_ [G] = 3.18 × *p*O_2_ [%] + 0.028, in selected region fidelity is *R*^2^ = 0.9996. Literature provides similar data, obtained after plot digitalization Δ*B*_pp_ [G] = 3.95 × *p*O_2_ [%] + 0.013,^24,25^ Δ*B*_pp_ [mG] = 6.84 × *p*O_2_ [%] + 0.009 (linear region up to 7.5% O_2_, saturation level at 0.6 G (ref. 39)), Δ*B*_pp_ [G] = 3.8 × *p*O_2_ [%] + 0.04,^6^ Δ*B*_pp_ [G] = 5.96 × *p*O_2_ [%] + 0.024 (ref. 40) and similar sensor based on naphthalocyanine (LiNc) Δ*B*_pp_ [G] = 23.4 × *p*O_2_ [%] + 0.93 (ref. 41) and the case of LiNc-BuO Δ*B*_pp_ [G] = 6.4 × *p*O_2_ [%] + 0.3,^42^ LiNc:PDMS Δ*B*_pp_ [G] = 0.185 × *p*O_2_ [%] + 1.14 (ref. 43) (unit conversion described in the Experimental section). Results show slightly different slopes and saturation dependence in regions 0–21% O_2_. This can be the problem of differences in synthesis protocols, crystalline size, amorphous fraction present, and mixtures of α and γ phases in the final sensor. The signal of the γ-phase gets broader and weaker with increased oxygen, and the initially weak/invisible background line (of the α-phase or defects) can become visible.

**Fig. 5:**
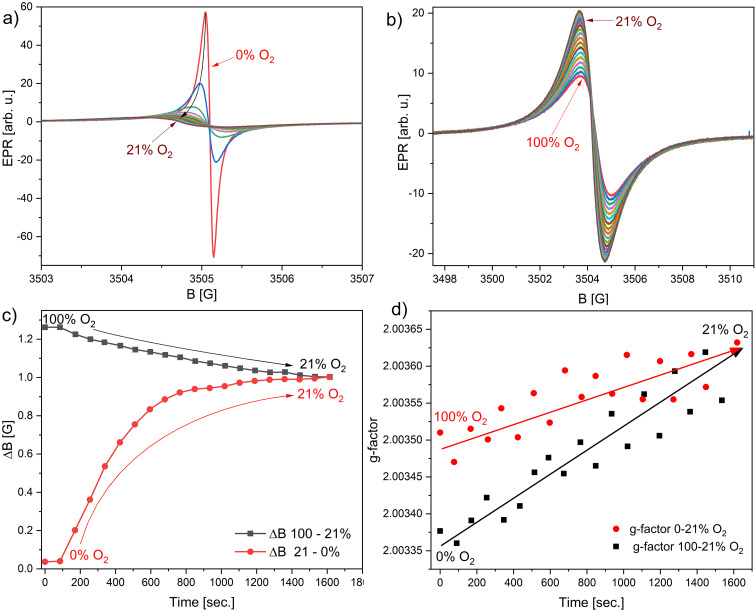
Successively collected EPR spectra, dwell time 100 ms of (a) nitrogen saturated γ-LiPc; (b) oxygen saturated γ-LiPc sample; (c) peak-to-peak linewidth changes in time during desaturation of the atmosphere with N_2_ (red circles) and second experiment with O_2_ (black squares); and (d) *g*-factor changes during experiment described in the case shown in Fig. 4c.

The temporal dependencies of peak-to-peak linewidth and *g*-factor are shown in Fig. 5. For technical reasons, linewidth dependencies were obtained starting from 0% O_2_, which is saturation with N_2_ gas, and the second experiment started from saturation with O_2_ gas. Fig. 5a shows the decrease in amplitude and increase of linewidth and *g*-factor during the rise of O_2_ concentration in the crystals. In Fig. 5b, the second part of the experiment is visible; starting from 100% O_2_ saturation, gas flow is stopped, signal amplitude increases and linewidth decreases, reaching stable air conditions of 21% O_2_. The *g*-factor changes are less than those observed during the previous experiment. Fig. 5c shows the dynamic change in the peak-to-peak linewidth during desaturation of the sample with N_2_ (red circles) and similarly for O_2_ (black squares). Transferring from 0% to 21%, the linewidth takes *ca.* 25 min. (points were recorded every *ca.* 80 s). Fig. 5d shows the changes in the *g*-factor, whereas the reference was the Al_2_O_3_:Cr^3+^ internal standard previously calibrated with the DPPH standard with an assumed *g*-factor equal to 2.0036. Gas flow was restricted after the first two points were recorded under saturation conditions. Both experiments meet at 21% O_2_ (air conditions) for a linewidth of 1.05 G and *g*-factor of 2.0036. Recorded changes in this parameter are small, 2.0033–2.0036. The lowest observable peak-to-peak linewidth depends on the synthesis batch (sample purity) and is in the 44–65 mG range. The largest measured linewidth is *ca.* 1.25 G (100% O_2_).

**Fig. 6:**
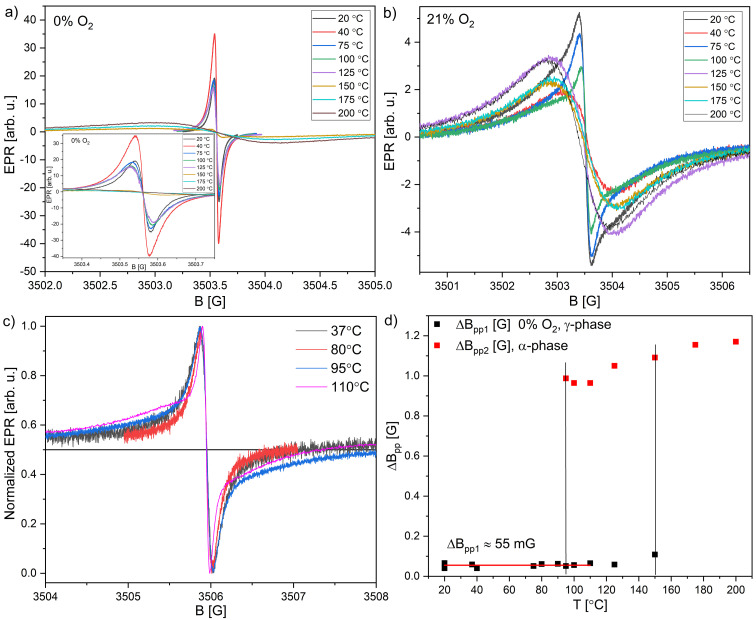
EPR linewidth *vs.* heating temperature (2 h) for (a) 0% O_2_ and (b) 21% O_2_; (c) selected EPR lines from a sample heated for 2 h at 37, 80, 95, 110 °C (21% O_2_); (d) collection of peak-to-peak linewidths of previously heated samples-red squares show the peak-to-peak linewidth of the component insensitive to oxygenation, which appears at 95 °C and black squares are the narrowest linewidths recorded for 0% O_2_. Above 150 °C, only a broad line exists, which is insensitive to oxygenation. Data is collected from multiple samples and averaged.

Fig. 6a and b show the EPR spectra of the selected sample measured the day after synthesis (20 °C sample) in 21 and 0% O_2._ Spectra were recorded after 2 h of heating in a dryer at temperatures 40–200 °C. In general, moderate drying (*T* < 75 °C) improves signal intensity due to the opening of the channels in crystals, getting out moisture, and synthesis residue. Fig. 6c shows the appearance of the additional broad component, which at 95 °C is barely visible in the wings of the signal, and then at 110 °C, it appears clearly. Previously, it has been reported that LiPc is stable in the temperature range of 0–200 °C.^7^ In this range, only the insensitive α-phase is stable, where the γ-phase transitions to α-phase is already at 95 °C (Fig. 6d). This process continues up to 150 °C, in which the amplitude of the insensitive-to-oxygenation component of the α-phase gains strength. In the range of 150–200 °C, only one broad component exists (α-phase), rendering the sensor useless. Due to EPR spectroscopy's sensitivity, the transition's beginning could be seen at lower temperatures than seen using XRD.

### Electrochemical impedance spectroscopy (EIS) and cyclic voltammetry

**Fig. 7:**
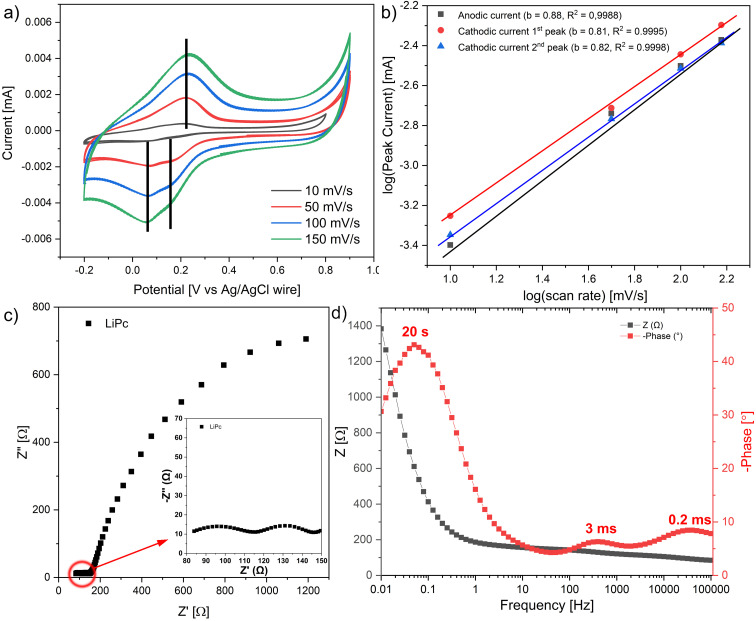
(a) Cyclic voltammetry of LiPc film with scan rates from 10 to 150 mV s^−1^ recorded with a three-electrode configuration, (b) dependence of the log(scan rate) *vs.* log(anodic or cathodic peak currents). (c) Nyquist plot of LiPc film (inset – high-frequency region), and (d) Bode plot of LiPc.

To investigate the electrochemical performance of the LiPc film, the LiPc film was deposited using the CV method in a three-electrode cell configuration. The detailed deposition and characteristic curves are presented in the ESI.† The CV curves of the LiPc film with the scan rates from 10 mV s^−1^ to 150 mV s^−1^ were recorded in the potential window from −0.2 to 0.9 V *vs.* Ag/AgCl wire (Fig. 7a). Only one broad anodic peak was recorded for all scan rates. However, two cathodic peaks were recorded, as presented in Fig. 7a. The overlapping of the two peaks for anodic current could explain the observation of only one broad anodic peak. Further analysis of the CV curves of LiPc film, particularly building a graph of the log(scan rate) *vs.* log(peak current), which is presented in Fig. 7b, confirms that the obtained LiPc film possesses the ability for oxidation and reduction that did not depend on the speed of the electrochemical reaction. The fitting of the dependences for anodic and two cathodic currents gave values of 0.88 and 0.81 or 0.82, respectively (Fig. 7b). These values for all peak current approaches to a value of 1 meaning that electrochemical processes (oxidations/reductions) are not diffusion-controlled. The peak potentials for both the anodic and the cathodic peaks were found not to increase with the sweep rate, a typical characteristic of the reversible redox process. The presence of one broad anodic peak and two cathodic peaks transform the cation-radical to the neutral form for the two LiPc phases: α-phase and γ-phase.

EIS provided complementary information to that obtained from cyclic voltammetry. The EIS was measured in the frequency range from 100 kHz to 0.01 Hz with 5 mV amplitude, and the results are presented in the form of Nyquist plots and Bode plots, Fig. 7c and d, respectively. The total impedance of the film does not change in the frequency range from 100 kHz to 1 Hz, as observed in Fig. 7d, with a total value of 150 Ω, which confirms that LiPc film is a semi-conducting material. The presence of α-phase and γ-phase in the LiPc film was observed from two small semicircles at a high-frequency range (presented in Fig. 7c insert). These semicircles correspond to the interaction of α-phase and γ-phase with the electrolyte, and the time of this interaction is in the ms time domain. The large semicircle at the low-frequency range corresponds to the double-layer formation of LiPc film.

The general conclusion from the cyclic voltammetry and EIS is that both methods confirmed the presence of two phases: α-phase and γ-phase. Both phases have similar redox properties, and charge–transfer processes occur in the ms time domain region. However, it was impossible to distinguish which process corresponds to α-phase and which one to γ-phase.

## Conclusions

The γ-phase of LiPc is sensitive to oxygenation owing to its unique structure-open channels of *ca.* 6 Å diameter that allow O_2_ molecules to penetrate. The LiPc response on O_2_ molecules is linear in the biologically relevant range and reversible, confirming it has physisorption and not chemical bonding. Linewidth response after rapid oxygenation changed from 0–21% takes *ca.* 25 min. Line width dependence on oxygen was linear up to 21% partial pressure. Above this value, linewidth increased, and saturation effects were visible. The O_2_-sensitive γ-phase is stable in lower temperature ranges than previously stated in the literature. The insensitive α-phase appeared at 95 °C, and its intensity increases at the cost of the first one. Only the α-phase was present above 150 °C, which is insensitive to O_2_. This report is the first one on the surface zeta potential of both lithium phthalocyanine crystals. The two differ significantly, with −53.08 ± 2.37 mV for Li_2_Pc and −2.4 ± 0.61 mV for LiPc. The latter phase offered less irritation to the immune system, making it a more efficient and promising biosensor.

## Data availability

The data supporting this article have been included as part of the ESI.†

## Author contributions

Elena Tomsik – formal analysis, investigation, writing EIS and CV chapter. Zulfiya Cernochova – formal analysis, investigation, writing DLS chapter, writing – review & editing. Magdalena Scheibe – formal analysis, investigation, writing Raman scattering chapter. Krzysztof Tadyszak – sample preparation, formal analysis, investigation, writing EPR chapter, writing – review and editing.

## Conflicts of interest

The authors declare that they have no known competing financial interests or personal relationships that could have appeared to influence the work reported in this paper.

## Supplementary Material

RA-015-D4RA08335K-s001
