# Supplementary material for: Lithium phthalocyanine (γ-structure) as a molecular oxygen sensor
Source: RSC Adv. 2025 Feb 5;15(5):3738–48. doi: 10.1039/d4ra08335k (PMC11795808; doi:10.1039/d4ra08335k)
Supplement: RA-015-D4RA08335K-s001 [file RA-015-D4RA08335K-s001.pdf]

Supplementary Information (SI) for RSC Advances.
This journal is © The Royal Society of Chemistry 2025

Supplementary information

Lithium phthalocyanine (γ - structure) as molecular oxygen sensor

Authors: Elena Tomsik[1], Zulfiya Cernochova[1*], Magdalena Scheibe[2], Krzysztof Tadyszak[1*]

[1] *Institute of Macromolecular Chemistry, Czech Academy of Sciences, Prague, Czech Republic*
[2] *J. Heyrovsky Institute of Physical Chemistry, Czech Academy of Sciences, Prague, Czech Republic*

Correspondence: Krzysztof Tadyszak, email: *tadyszak@imc.cas.cz*; Zulfiya Cernochova, email: *cernochova@imc.cas.cz*

Table 1S The size distribution of obtained crystals.

Bin	Crystal length [μm]	Bin	Crystal width [μm]
0 - 1	32	0.1 - 0.2	15
1-2	204	0.2 - 0.3	89
2-3	103	0.3 - 0.4	144
3-4	35	0.4 - 0.5	125
4-5	13	0.5 - 0.6	62
5-6	2	0.6 - 0.7	34
6-7	1	0.7 - 0.8	8
7-8	1	0.8 - 0.9	2
8-9	0	0.9 - 1	1
9-10	0		
10-11	1		

Table 2S. Zeta potential measurements for Li_2Pc and LiPc water solutions at 25°C and 37°C degrees of the temperature.

No.	Li_2Pc			
	25°C		37°C	
	ZP, mV	ZP accuracy	ZP, mV	ZP accuracy
1	-52.6	0.75056	-42	0.32146
2	-54.1	0.49329	-42.5	0.05774
3	-53.3	0.15275	-42.6	0.51962
4	-53.2	0.15275	-42.6	0.45092
5	-53	0.37859	-43.5	0.40415
6	-52.9	0.32146	-43	0.35119
7	-52.3	0.15275	-42.7	0.65064
8	-52.4	0.41633	-43.4	0.78102
9	-52.6	0.41633	-42.1	0.05774
10	-51.8	0.34641	-42	0.11547

11	-52.4	0.34641	-42	0.52915
12	-51.8	0.41633	-41.8	0.4
13	-52.4	0.2	-41	0.23094
14	-52.6	0.14142	-41.4	0.4
15	-52.8	0.2	-41.4	0.23094
	LiPc			
	25°C		37°C	
1	-0.528	0.16302	-5.65	0.17898
2	-0.528	0.16302	-5.96	0.25697
3	-0.318	0.06409	-5.65	0.30665
4	-0.207	0.06092	-6.16	0.03055
5	-0.318	0.00603	-6.2	0.3581
6	-0.306	0.0265	-6.22	0.55194
7	-0.313	0.10593	-5.59	0.57327
8	-0.264	0.23312	-6.69	0.50718
9	-0.467	0.21533	-5.86	0.57622
10	-0.729	0.26317	-5.77	0.62482
11	-0.302	0.06643	-6.81	0.05686
12	-0.249	0.09403	-6.89	0.57709
13	-0.381	0.02532	-6.78	0.47286
14	-0.431	0.16306	-5.84	0.1914
15	-0.413	0.20577	-6.22	0.10607
16	-0.704	0.16306	-6.07	0.1914

Table 3S g-factors vs. oxygen concentration in O_2/N_2 gas mixture.

100-21 %O_2	g-factor	0-21 %O_2	g-factor
0	2.00338	0	2.00351
90.347	2.00336	75.869	2.00347
170.27	2.00339	166.216	2.00352
253.668	2.00342	260.039	2.0035
347.49	2.00339	333.012	2.00354
434.363	2.00341	423.359	2.0035
514.286	2.00346	510.232	2.00356
590.734	2.00348	597.104	2.00352
674.131	2.00345	680.502	2.00359
764.479	2.0035	770.849	2.00356
847.876	2.00347	847.297	2.00359
934.749	2.00354	937.645	2.00356
1021.622	2.00349	1017.568	2.00362
1111.969	2.00356	1104.44	2.00356
1195.367	2.00351	1198.263	2.00361
1278.764	2.00359	1271.236	2.00356
1362.162	2.00354	1368.533	2.00362
1445.56	2.00362	1448.456	2.00357
1535.907	2.00355	1618.726	2.00363
1608.88	2.00362		

Table 4S Dynamic change of linewidth after abrupt change of oxygen concentration.

Time [sec.]	ΔB [G] 21-100% O_2	Time [sec.]	ΔB [G] 0-21% O_2
0	1.26211	0	0.102
85.098	1.26211	85.098	0.10379
172.508	1.22496	169.995	0.20211
257.416	1.1996	254.874	0.36166
337.2522	1.18344	339.746	0.53604
427.186	1.16578	424.63	0.66075
512.12	1.14535	509.516	0.75467
597.021	1.13371	594.37	0.83359
681.951	1.11777	679.267	0.88564
766.853	1.10531	764.164	0.92136
851.74	1.08508	849.017	0.93944
936.664	1.07377	933.888	0.94482
1021.545	1.06076	1018.772	0.95442
1106.449	1.04795	1103.665	0.97271
1191.386	1.03646	1188.578	0.98191
1276.261	1.02657	1273.439	0.98832
1361.162	1.02784	1358.313	0.99127
1446.03	1.01182	1443.227	0.98973
1530.918	1.00406	1528.11	0.99501
1615.836	1.00221	1612.988	1.00223

Table 5S EPR linewidth change after drying.

Temp. [^{o}C]	ΔB [G]	ΔB [G]
20	0.04	
20	0.066	
37	0.059	
40	0.04	
75	0.0511	
80	0.061	
90	0.062	
95	0.051	0.98744
100	0.0558	0.96406
110	0.065	0.96406
125	0.0586	1.05003
150	0.1086	1.09123
175		1.15461
200		1.17

Table 6S Dependence of the log(scan rate) vs log(anodic or cathodic peak currents).

log (scan rate) [mV/s]	log (Peak Current) [mA]	log (Peak Current) [mA]	log (Peak Current) [mA]
1	-3.39794	-3.25181	-3.34679
1.69897	-2.73993	-2.7122	-2.767
2	-2.50169	-2.4437	-2.51428
2.17609	-2.37161	-2.29671	-2.38722
